# Popular Physiology.—The Nervous System
**The Physiology of Common Life:* By George Henry Lewes. 2 vols. Blackwood and Sons, 1860. Vol. II., *The Nervous System*.


**Published:** 1860-07-01

**Authors:** 


### Art. VII.—POPULAR PHYSIOLOGY.—THE NERVOUS SYSTEM*

Mr. George Henry Lewes is an enthusiastic naturalist and physiologist, and one of the most brilliant writers of the day on scientific subjects. He has so happy a gift with the pen that he charms you even when what he advances is directed in the teeth of your most settled convictions. He treads without compunction on your tenderest opinions, and yet compels you to simper and smile the while. In the last of Mr. Lewes's fully published works, he, for example, as well in the conception as in the execution of the book, dashes right through old-standing ideas both of the method and manner of popular instruction. Still the book is so delightful to read, so full of admirably told information, that we can heartily sympathize with that great pleasure with which it has been received on all hands, and we have little doubt that it will long hold its own as the most popular of popular works on physiology.

"No scientific subject can be so important to man as that of his own life," wrote our author in the announcement which heralded his work; "no knowledge can be so incessantly appealed to by the incidents of every day, as the knowledge of the *processes by which he lives and acts.* At every moment he is in danger of disobeying laws which, when disobeyed, may bring years of suffering, decline of powers, premature decay. Sanitary reformers preach in vain, because they preach to a public which does not understand the laws of life—laws as rigorous as those of gravitation or motion. Even the sad experience of others yields us no lessons, unless we understand the *principles* involved. If one man is seen to suffer from vitiated air, another is seen to endure it without apparent harm; a third concludes that 'it is all chance,' and trusts to that chance; had he understood the principle involved, he would not have been left to chance—his first lesson in swimming would not have been a shipwreck."

This is an old and familiar story, but not the less true because familiar. Well did Phineas Fletcher sing in his *Purple Island, or the Isle of Man,* and well might Mr. Lewes sing after him :—

* *The Physiology of Common Life:* By George Henry Lewes. 2 vols. Blackwood and Sons, 1860. Vol. II., *The Nervous System.*

"Hark then, ah, hark! yon gentle shepherd cried;
An isle I fain would sing, an island fair,
A place too seldom view'd, yet still in view;
Near as ourselves, yet farthest from our care;
Which we by leaving find, by seeking lost;
A foreign home, a strange, tho' native coast;
Most obvious to all, yet most unknown to most.

"Coeval with the world in her nativity,
Which tho' it now hath pass'd thro' many ages,
And still retain'd a natural proclivity
To ruin, compassed with a thousand rages
Of foemen's spite, which still this island tosses,
Yet ever grows most prosp'rous by her crosses,
By with'ring springing fresh, and rich by often losses.

"Vain men, too fondly wise, who plough the seas,
With dang'rous pains another earth to find;
Adding new worlds to th' old, and scorning ease,
The earth's vast limits daily more unbind!
The aged world, though now it falling shows,
And hastes to set, yet still in dying grows:
Whole lives are spent to win what one death's hour must lose.

"Yet this fair isle, seated so nearly near,
That from our sides, nor place, nor time, may sever;
Though to yourselves yourselves are not more dear,
Yet with strange carelessness you travel never:
Thus while yourselves and native home forgetting,
You search for distant worlds, with needless sweating.
You never find yourselves; so lose ye more by getting."*

No doubt if the general public were possessed of a better knowledge of the laws of life, they might listen with more respect to the teaching of sanitary reformers. No doubt the numerous and distinguished physicians and surgeons were right, who in 1853 presented an opinion to the Government, respecting tuition in common schools, which terminated thus:—"We are therefore of opinion that it would greatly tend to prevent sickness, and to promote soundness of body and mind, were the elements of physiology, in its application to the preservation of health, made a part of general education; and we are convinced that such instruction may be rendered most interesting to the young, and may be communicated to them with the utmost facility and propriety in the ordinary schools by properly instructed schoolmasters." We shall not question this conclusion, but it has always appeared to us to hold a very similar position to the dogma that in order

* Canto i.

to know practical religion it is necessary first to be indoctrinated in theology, properly so called. Sanitary science is, indeed, a science which has its own data to build upon. For the explanation of these, both physiological and physical science must be had recourse to. The certainty of the explanation in the majority of instances by no means corresponds with the certainty of the data to be explained. Assuredly, therefore, our teaching should invariably begin with what is most certain. Now the physiology actually available in the teaching of sound sanitary rules is certainly not very extensive, and it always and invariably finds a legitimate place in the teaching of those rules. To start, therefore, from physiology to teach sanitary science, is to endeavour needlessly to educe practical certainties from speculative uncertainties; and to dignify the physiological items which may be made use of for the explanation, confirmation, and right appreciation of sanitary facts and rules, whether applicable to the individual or communities, with the term *physiology*, is a misnomer.

We have little doubt that to the profuse and unguarded use of the phrase *physiology* to designate the items of that science, which under the guise of elementary and rudimentary treatises have too often found their way into our school-rooms and libraries, we are indebted in no small degree for those pseudo-scientific follies which have abounded of late. The innocent imbibers of these crude treatises are but too often puffed up with the vain imagination that they have laid a solid foundation of physiological information, and are prepared to dogmatize upon any question which may arise in reference to man, mental or physical. They have been furnished with sundry stray tools, it matters not whether good or bad, and they have not been taught how to use those tools, but have been led to believe that because they have them in possession, therefore they are qualified to use them. This is the great vice of the popular teaching of the present day on almost all scientific subjects.

But sanitary science being, as we assert, a science resting upon specific data, it is a curious fact that while the importance of making its principles a portion of the education of the people at large is being recognised, yet these principles are only taught incidentally, so to speak, to the very men from whom the public would chiefly have to learn, that is to say, medical men. With one or two exceptions, sanitary science is not taught in any of the medical schools of this country. Moreover, there is not a complete textbook, or book of any kind, on the subject in the English language.* If we do not err the only specific lectureships on sanitary science

* Dr. Pickford, of Brighton, published the first part of a work on *Hygiène* in 1858, but the work, so far as we are aware, has not been completed.

in London are in the medical schools of the St. Thomas's Hospital and of Grosvenor-street. But the attendance upon the lectures delivered upon this subject is *optional* to the students. We would commend this strange anomaly in our tuition of medicine to the members of the National Association for the Promotion of Social Science. We hold that any attempt to teach effectively sanitary science to the people will prove abortive, until medical men, who must, in the majority of instances, be the chief authorities referred to in doubtful questions, be themselves disciplined in the science as a science.

But in addition to the importance attached to the popular teaching of physiology as a means of promoting a knowledge of sanitary science among the people, it is frequently urged that the former would contribute, even in common schools, to a better intellectual training. We doubt this. Dr. Whewell has said that—

"No ideas are suited to become the elements of elementary education, till they have not only become perfectly distinct and fixed in the minds of the leading cultivators of the science to which they belong; but till they have been so for some considerable period. The entire clearness and steadiness of view which is essential to sound science, must have time to extend itself to a wide circle of disciples. The views are principles which are detected by the most profound and acute philosophers; are soon appropriated by all the most intelligent and active minds of their own and of the following generations; and when this has taken place (and not till then), it is right, by a proper constitution of our liberal education, to extend a general knowledge of such principles to all cultivated persons. And it follows, from this view of the matter, that we are by no means in haste to adopt, into our course of education, all new discoveries as soon as they are made. They require some time, in order to settle into their proper place and position in men's minds, and to show themselves under their true aspects; and till this is done, we confuse and disturb, rather than enlighten and unfold, the ideas of learners, by introducing the discoveries into our elementary instruction."*

Agreeing entirely with Dr. Whewell in these opinions, it will readily be understood that we cannot avoid questioning the beneficial effects of Mr. Lewes's work in an educational point of view, looking upon that work in the light indicated by himself. He tells us that its object "differs from all other works on popular science in its attempt to meet the wants of the student, while meeting those of the general reader, who is supposed to be wholly unacquainted with anatomy and physiology." This in the preface. Elsewhere he writes:—"Assuming the position of a lecturer addressing a miscellaneous audience, he will imagine that beside the Medical Student there sits an intelligent Artisan,

* *Novum Organon Renovatum*, p. 178.

beside the Man of Letters sits the Mother of a family; and he will endeavour to be intelligible and interesting to all, while reproducing the *latest* discoveries of European investigators, and the results of *original* research." Then, again, Mr. Lewes writes, in the preface, of the manner of executing the work:—

"In pursuance of this object I have been forced to depart very widely from the practice of other popular writers, who consider themselves bound to act as 'middle-men' between scientific authorities and the public, and to expound facts and doctrines as they find them. I could not adopt this easy and convenient plan. I could not bring myself to publish, on the authority of respected names, statements which I knew to be false, and opinions which I believed to be erroneous. After having laboured earnestly to get at the truth, it would have been disloyal to contribute in any way to the spread of what I believed to be error. All that I felt bound to do, was to state impartially the facts and opinions current among physiologists; and, when those opinions seemed inadmissible, to state the reasons for their rejection. There is therefore a great deal of criticism, and much original matter in this work."

Now the portion of Mr. Lewes's work which most concerns us is that devoted to the Nervous System, and it is in this portion that is found, as he correctly observes, "the greatest amount of dissent from correct opinions;" and it is there that the reader will have the "greatest difficulty in agreeing with him." So great is this difficulty on our own part, that we find our objections and our doubts running tolerably evenly side by side with his peculiar conclusions, and to do justice to either one or the other it would be requisite to follow Mr. Lewes's opinions *seriatim*. This, however, we need not do, as a few hints will suffice for every useful purpose.

And first of the nerves: are they conductors only? and what do they conduct? Mr. Lewes writes:—

"Nerves are very generally likened to telegraphic wires, carrying messages to and from the centres; or to the conducting wires of a galvanic battery. But except as a loose and superficial analogy, this is not acceptable; and it is based on a misconception so important, that we must pause a moment to consider it. The misconception is, that the centres *produce* a force which the nerves, as passive conductors, *transmit*. The analogy is to the plates of a battery producing the electricity, which the wires conduct. I think there is ample and decisive evidence to show that the nerves have a force of their *own*, the property of their tissue, which is far from being the product of nerve-centres, and is wholly *unlike* that produced by the centres." (Vol. ii., p. 14.)

Further, Mr. Lewes remarks:—

"When the conducting wire is separated from the battery, it loses at once all galvanic power; it is a bit of wire, and it is nothing more. All its galvanism came from the battery; and this it could only conduct, not create. But the nerve, when separated from its centre, still retains its force; any irritation of such a nerve will excite that force, just as the stimulus from its centre would excite it. The battery is removed, and lo! the wire is found to be galvanic." (p. 18.)

Now these being Mr. Lewes's notions of galvanic action in a "conducting wire," his previous remarks admit of some justification. But the said notions being by no means such as we have been accustomed to hold (following Faraday) respecting the transmission of galvanic force, and such as may be presupposed to be held, and which so far as we know are held, by our leading physiologists, Mr. Lewes's observations do not apply to their opinions, when they may analogically express the action of a nerve to that of a so-called "conducting wire." So that when Mr. Lewes assumes that nerves have a force of their own, which he proposes to call *Neurility* ("in the sense of *excitability*, but without the misleading suggestions of that word"); and further explains that "Neurility simply means the property which the nerve-fibre has, when stimulated, of exciting *contraction* in a muscle, *secretion* in a gland, and *sensation* in a ganglionic centre;" we do not see what we gain either by the new name, or the definition. It seems to us, that both the one and the other leave the facts which show the mode of action of nerves, and the expressions which have been made use of to convey a notion of that mode, just where Mr. Lewes found them, neither adding to the one, nor making apparent the unfitness of the other.

Mr. Lewes holds that "Sensibility is the *property inherent in ganglionic tissue*." Subsequently he writes: "Sensibility is ascribed to the ganglionic substance of the brain, or some portion of the brain, and denied to other masses of ganglionic substance, absolutely identical in all the fundamental characters. Nevertheless, no physiologist, to my knowledge, has been aware of this violation of a first principle."—(p. 22). Again: "It is simple logic, therefore, to conclude that there being one *common tissue*, there must be one *common property*, in brain, medulla, and chord, however various the *functions*, or uses, to which the property may in each case be applied. Experiment clearly verifies what logic thus deductively concludes—namely, that the spinal chord is in all animals a seat of sensibility; and in some animals the all important seat."—(p. 23). It is evident that Mr. Lewes is here using the term sensibility in a sense very different from most physiologists. Thus Müller says of sensibility, "It is only one among several functions of the nervous system. It would be an abuse of words to extend this denomination to functions unaccompanied by perception." Now, as this fact of perception is the determining element in the chief common, and the more persistently scientific use of the word, and has governed the application of the word even in its widest metaphorical sense, of what scientific utility can be the following generalization? "*Sensibility* is the property of ganglionic substance, and however various the uses or functions which different centres may serve—those of Respiration being very different from those of facial Expression, and these again from those of Perception, and so on—the same fundamental character is found in all"—(p. 24). Thus, then, Mr. Lewes would apply to the peculiar capacity of action possessed by ganglionic substance generally a term which it is customary to apply, and which can be definitely restricted to a definite mode of action of that substance in certain nervous centres. We cannot conceive that such an application of a term can add any clearness to our apprehension of the phenomena, the "fundamental characters" of which are sought to be generalized; neither can we conceive how a term, which in its most legitimate sense implies perception, can, without confusion, be applied scientifically to phenomena which are not supposed under any circumstances to be accompanied by perception.

Once more; Mr. Lewes tells us that:—

"One of the principal conclusions to which fact and argument will direct us in these pages will be, that the brain is only *one* organ of the mind, and not by any means the exclusive centre of Consciousness (p. 4) . . . It will be understood that by the word Mind, we do not designate the intellectual operations only. If the term were so restricted, there would be little objection to our calling the brain the organ of the mind. But the word Mind has a broader and deeper significance; it includes all sensation, all volition, and all thought; it means the whole psychical life, and this psychical life has no one special centre any more than the physical life has one special centre; it belongs to the whole, and animates the whole. The brain is a part of this whole, a noble part, and its functions are noble; but it is only the organ of special mental functions, as the liver and the lungs are organs of special bodily functions. It is a centre, a great centre, but not *the* centre. It is not the exclusive sensorium. Its absence does not imply the absence of *all* consciousness, as I shall prove by experiment. It cannot, therefore, be considered as *the* organ, but only as *one* organ of the mind." (p. 5.)

Here then, as in the case of the word sensibility, Mind and Consciousness are used in a sense very different to that in which they are commonly used. They are made to include phenomena usually expressed by different terms.

Let us briefly examine an illustration of the application of this doctrine and that of sensibility. We take it from the section on the evidence against the sensibility of the spinal chord. Mr. Lewes admits:—

"That injury to the spinal chord wholly or partially destroys the power of obeying the Brain by voluntary movement, and the power of transmitting sensory impressions to the Brain, in the parts *below* the seat of injury; while in those *above* the seat of injury, sensation and voluntary motion remain.

"Such is the conclusion rigorously deduced from numerous facts. I accept it, without reserve. But I shall now prove that it does not in the least affect the question under discussion, does not throw a shadow of doubt on the sensational and volitional character of the spinal chord. That it should ever have been thought to do so admits of easy explanation. Let us disclose the fallacy it involves.

"On the supposition that the whole cerebro-spinal axis is everywhere the seat of sensibility, it has already been shown that division of this axis would create two independent centres. In this case we have no right to suppose that the *cerebral* segment will be affected by impressions made on the *spinal* segment; nor, conversely, that impressions made on the cerebral segment will affect the spinal segment. The anterior limbs will obey the brain, because they are in organic relation with it; but the posterior limbs *cannot* obey the brain after they have ceased to be in organic relation with it. This has been fully explained (p. 249 *et seq.*)

"Now, when a man has a diseased spinal chord, the seat of injury causes, for the time at least, a division of the cerebro-spinal axis into two independent centres. For all purposes of sensation and volition it is the same as if he were cut in half; his nervous mechanism *is* cut in half. How, then, can any cerebral volition be obeyed by his legs; how can any impression on his legs be felt by his cerebrum? As well might we expect the man whose arm has been amputated, to feel the incisions of the scalpel, when that limb is conveyed to the dissecting-table, as to feel in his brain impressions made upon parts wholly divorced from organic connexion with the brain.

"But, it may be objected, this is the very point urged. The man himself does not feel the impressions on his limbs when his spine has been injured; he is as insensible to them as to the dissection of his amputated arm. Very true. *He* does not feel it. But if the amputated arm were to strike the anatomist who began its dissection, if its fingers were to grasp the scalpel, and push it away, or with the thumb to rub off the acid irritating one of the fingers, I do not see how we could refuse to admit that the *arm* felt although the *man* did not. And this is the case with the extremities of a man whose spine is injured. *They* manifest every indication of sensibility. In the frog they manifest unmistakable volition. It is true that the man himself, when interrogated, declares that he feels nothing; the cerebral segment has attached to it organs of speech and expressive features, by which *its* sensations can be communicated to others; whereas the spinal segment has *no* such means of communicating *its* sensations; but those which it *has*, it *employs*. You can ask the cerebral segment a question, which can be heard, understood, and answered; this is not the case with the spinal segment; yet if you *test* its sensibility, the result is unequivocal. You cannot ask an animal whether it feels, but you can test its sensibility, and that test suffices."

Now, even admitting the correctness of Mr. Lewes's conclusions from his experiments on the effects of section of the spinal chord of the frog, referred to in the preceding paragraphs, what advantage can arise from applying, in a scientific sense, terms of which we can only know and apprehend the signification as referring to the man in his entirety, to circumstances in which the terms must bear an entirely different signification? Must not such a course inevitably lead to confusion, by widening illimitably, and, indeed, rendering incongruous, the meaning of words which at the present time have tolerably well-defined common acceptations which can be, and are usually, restrained within strictly scientific bounds?

There are certain observations of Mr. Mansel's on "an unacknowledged anthropomorphism" that pervades our speculations on consciousness which it may not be inutile to quote here. Mr. Lewes uses consciousness as an equivalent term to sensibility, and we have seen how greatly he extends the meaning of the latter word. The following remarks of Mr. Mansel, on a portion of Professor Ferrier's theory of Knowing and Being, we think clearly indicate the inadvisability of such an extension of the signification of the word consciousness as Mr. Lewes proposes, and points out the source of much error in the use of that word. Mr. Mansel writes:—

"Let us try Professor Ferrier's theory in three special instances, selecting that portion of his axiom, ['Along with whatever any intelligence knows, it must, as the ground or condition of its knowledge, have some cognizance of itself:'] which, as limited to human consciousness, is unquestionably true.

"1. Whatever state of consciousness I experience, I must know that state as mine.

"2. Whatever state of consciousness an angel experiences, he must know that state as his (the angel's).

"3. Whatever state of consciousness an oyster experiences, he must know that state as his (the oyster's).

"Are these three statements equally self-evident? Most people at first sight would admit the first and second, but doubt about the third. How do we know, they might ask, that the oyster has an idea of self at all? How do we know that he has memory; that he can associate one sensation with another, and know himself as the subject of all? Why, then, are we most confident about the angel, in whose case we have no more warrant of experience than in that of the oyster? Simply because we can subtract from the sum total of our own consciousness, but cannot add to it. I think of a lower intelligence as a part only of my own; and I see that the subtraction may possibly change the entire result. I think of a higher intelligence as my own, and something more; but, this something being totally unknown, I assume, quite gratuitously, that it will not interfere with the poorest operations of the remainder. Hence I have no difficulty in anthropomorphizing the angel; but I do not find it so easy to anthropomorphize the oyster. Where my own intelligence is but a part, I am well content to reason as if it were the whole; but where it is the whole, I am not equally ready to identify it with the part. But I have not thereby advanced one step in the knowledge of the conditions of other than human intelligences. I have only made my own intelligence the representative of all. I have generalized the *Ego*, and named it Pan: I have gazed on the image of my own mind, and in the microcosm I have symbolized the Universe."*

In quoting this psychological fragment, we are running counter to the canons of criticism which Mr. Lewes has enjoined for his book. He holds that the nervous system "must be studied free from all control on the part of psychologists. If we (the physiologists) do not prescribe conclusions for them, neither must they prescribe conclusions for us." He would, therefore, consider the psychologist "as out of court"—(p. 3). If we have broken our author's precepts, it has assuredly been because we have not been able clearly to recognise the line of demarcation he would have us heed. But here we terminate our fragmentary criticism. The illustrations we have given, showing how greatly we differ from Mr. Lewes in the significations we attach to certain words of very frequent occurrence in any dissertation of the nervous system, might have been added to if it were needful. But we shall not multiply instances. Sufficient has been said to indicate that our position with regard to several of Mr. Lewes's views on the nervous system is pretty much that of the logicians in the celebrated recital of Sawkenbergius:—

"... The logicians stuck much closer to the point before them than any of the class of the *literati;* they began and ended with the word *nose;* and had it not been for a *petitio principii,* which one of the ablest of them ran his head against in the beginning of the combat, the whole controversy had been settled at once.

"'A nose,' argued the logician, 'cannot bleed without blood—and not only blood, but blood circulating in it to supply the phenomenon with a succession of drops (a stream being but a quicker succession of drops, that is included, said he). Now death,' continued the logician, 'being nothing but the stagnation of the blood——'

"'I deny the definition. Death is the separation of the soul from the body,' said his antagonist. 'Then we don't agree upon our weapons,' said the logician. 'Then there is an end of the dispute,' replied the antagonist."

The chapters on the nervous system, although of chief interest to us, barely exceed a third of Mr. Lewes's work, every portion

* *Psychology the Test of Moral and Metaphysical Philosophy:* an Inaugural Lecture. By Henry Longueville Mansel, B.D., Reader in Moral and Metaphysical Philosophy, Magdalen College, Oxford. 1855, p. 43.

of which possesses that rare charm which he has the gift of infusing into all his writings. It is very far from requisite that we should attempt to whet the appetites of our readers by any samples which might convey a notion of the attractive qualities of the book, for the work must be already well known to them. Rarely, indeed, if common report be true, has a treatise on physiology been so widely read; rarely have we closed a book which, while embodying so much from which we dissent, has so largely excited our admiration as the *Physiology of Common Life.*

---

